# Synergistic Combination of Additive One‐ and Two‐Photon Polymerization Printing Methods to Fabricate 3D Microstructured Perfusable Angiogenesis–on–a–Chip Systems

**DOI:** 10.1002/elsc.70059

**Published:** 2026-01-27

**Authors:** Daria Sokoliuk, Rizlene Bouhaya, Peter Haeger, Kathrin Godthardt, Daniel Fetting, Lenard Spiecker, Heinrich Spiecker, Alexander Rockenbach, Holger Rothe, Klaus Liefeith, Doris Heinrich

**Affiliations:** ^1^ Miltenyi Biotec B.V. & Co. KG Bergisch Gladbach Germany; ^2^ Faculty of Mathematics and Natural Sciences Technische Universitaet Ilmenau Ilmenau Germany; ^3^ Institut Für Bioprozess‐ und Analysenmesstechnik e.V. (iba) Heilbad Heiligenstadt Germany

**Keywords:** biofunctionalization, microfluidics, organ‐on‐a‐chip, stereolithography, two‐photon polymerization

## Abstract

Tissue engineering, and in particular the development of organ‐on‐a‐chip (OOC) models, holds significant promise for advancing personalized medicine and reducing the use of animal models. The integration of microfluidics and advanced biomaterials in OOC systems provides controlled microenvironments and fosters the creation of physiologically relevant tissue models. A critical aspect of OOC models is the fabrication of perfusable chips to create vascular networks that are essential for sustaining long‐term 3D cultures. Here we show a two‐step fabrication approach that combines one‐ and two‐photon polymerization (2PP) to create a microfluidic chip capable of supporting endothelial cell (EC) angiogenesis. The chip features a 2PP‐printed sealing contour to ensure leak‐free bonding of chip parts, and an array of channel‐separating‐pillars that enable EC migration from the parent vessel into an extracellular matrix. Our results demonstrate that the developed angiogenesis‐on‐a‐chip model successfully induces EC sprouting in response to angiogenic factors. This work significantly contributes to the field by providing a versatile platform for vascular studies, highlighting the potential for its application in drug screening. The flexibility and precision of our fabrication method also allows for customizing OOC devices for various biological applications, thereby enhancing the relevance of these systems in investigation of complex tissue interactions.

**Abbreviations**
2PP: two‐photon polymerization
AFs: angiogenic factors
BA740: 2,5‐bis[4‐[N,N‐bis‐[2‐(acetyloxy)ethyl]phenyl]‐methylene]‐(2E,5E)‐cyclopentanone
ECM: extracellular matrix
ECs: endothelial cells
GFP: green fluorescent protein
HUVECs: human umbilical vein endothelial cells
LCM: poly‐((D,L)‐lactide‐co‐ε‐caprolactone) dimethacrylate
OOC: organ‐on‐a‐chip
PBS: phosphate‐buffered saline
RT: room temperature
SLA: stereolithography

## 1 Introduction

Tissue engineering is an interdisciplinary research area with great potential for repair and replacement of damaged tissues, and for modeling of human physiology and pathophysiology including the study of pathomechanisms [1]. The combination of tissue engineering with advancements in microfluidics and in biomaterials results in the development of organ‐on‐a‐chip (OOC) models with high physiological relevance and complexity. Such microfluidic systems allow for precise control over experimental conditions [2] and minimize the use of animal models [3]. The integration of OOC systems with personalized medicine approaches enables more precise disease modeling and drug screening, leading to improved patient outcomes and the acceleration of drug development [4].

Fabrication methods play a pivotal role in the development and optimization of OOC systems, as they dictate the resolution, complexity, and reproducibility of the microfluidic structures embedded within these devices, as well as the usability of the OOC system. Among all varieties of fabrication techniques, light‐based methods gained considerable attention due to their high precision, scalability, and versatility [5, 6].

Two‐photon polymerization (2PP) is currently the most precise method for the additive manufacturing of complex three‐dimensional structures. The process is based on the quasi‐simultaneous absorption of two or more photons by a photoreactive molecule, typically a photoinitiator, which induces a polymerization reaction within a photoresist. The phenomenon of multiphoton absorption was first theoretically described by Maria Goeppert‐Mayer in 1931 [7], and the first practical demonstration was conducted in 1996 by Kawata et al. [8]. Two‐photon absorption is a nonlinear process, activated only at the focal point of a tightly focused laser beam. This enables 2PP to achieve resolutions far below the diffraction limit. Advances in the development of novel ultrashort‐pulse laser sources, combined with precise control in the photoresist via technologies such as Galvano scanners, have made 2PP a commercially viable rapid prototyping method applicable in various fields [9].

This unique characteristic enables direct 3D printing within the material, eliminating the need for layer‐by‐layer application [10]. In tissue engineering, 2PP is most commonly used for the fabrication of scaffolds that mimic the hierarchical structure of the extracellular matrix (ECM), supporting cell growth and tissue development [10, 11]. Applications include creating retinal cell grafts [12], vascular networks [11], engineering neural tissue [13], developing implants for bone [14, 15], cartilage [10, 15] regeneration and many more.

To achieve precise and customizable fabrication of microfluidic chips, we developed a two‐step fabrication approach that uses high resolution 2PP technology to manufacture application‐relevant microstructures inside a one‐photon (stereolithography, SLA) pre‐manufactured OOC system. This approach ensures high‐resolution features designed to meet specific user requirements, providing a versatile platform for various biological applications.

The introduction of photosensitive polymers used for the fabrication of OOC models through photopolymerization is crucial for successful tissue engineering. Chemically, the formulations used should exhibit desired IR transparency, viscosity, and polymerization efficiency. The printed structures should have minimal shrinkage or swelling. Moreover, as the material is intended for biomedical applications, it should also demonstrate biocompatibility, mechanical stability suitable for the chosen application, and controlled degradation at least if therapeutic applications are addressed. A good example of such a material is poly‐((D,L)‐lactide‐*co*‐ε‐caprolactone) dimethacrylate (LCM), a synthetic polymer with precisely defined biophysical properties, making it ideal for 2PP‐printing of scaffolds for tissue engineering. This material is versatile, supporting a wide range of applications from soft tissue models, such as breast cancer tumor scaffolds, to bone reconstruction, which demands significantly higher mechanical stiffness [16].

Importantly, vascular networks are essential for the maintenance and function of tissues and organs, providing vital support through the delivery of oxygen, nutrients, and signaling molecules [17]. Creating vascular networks within microfluidic chips is particularly important for the long‐term culture of 3D cell aggregates, which more accurately mimic human physiology as compared to 2D models [18]. Here, we focus on the first step in the direction of perfusable spheroids and organoids on the chip—the vascular structures themselves, and establish a 3D human cell‐based vascular network on a microfluidic chip. Complex vessel‐on‐a‐chip systems rely on multiple cell types, such as endothelial cells (ECs), pericytes, smooth muscle cells, and fibroblasts, to create physiologically relevant in vitro models [19]. Our system is designed to generate vascular structures for tissue perfusion, rather than modeling blood vessels, making a single cell type sufficient. We utilized human umbilical vein endothelial cells (HUVECs) tagged with green fluorescent protein (GFP), enabling easy real‐time visualization of vascular development.

In this work, we created an angiogenesis‐on‐a‐chip system, incorporating 2PP‐printed microstructures such as an array of pillars separating the channels, and a sealing contour protecting them. The function of the triangular pillars was enhanced by adding a channel separating border on the glass side, ensuring precise filling of the chip with an artificial ECM and enabling the migration of ECs through the ECM.

We demonstrated that our angiogenesis‐on‐a‐chip model is capable of sustaining ECs and inducing angiogenic sprouting in response to stimulation with angiogenic factors (AFs). Several experiments were conducted to validate the design and performance of the microfluidic chip. First, the biocompatibility of the 2PP‐material was assessed using GFP‐HUVECs cultured on discs containing LCM‐BA740 (precursor poly‐((D,L)‐lactide‐*co‐ε*‐caprolactone) dimethacrylate supplemented with the photoinitiator 2,5‐bis[4‐[N,N‐bis‐[2‐(acetyloxy)ethyl]phenyl]‐methylene]‐(2E,5E)‐cyclopentanone). The LCM‐BA740 was biofunctionalized with fibronectin, which enhanced EC adherence in comparison to uncoated samples. In the following experiments, GFP‐HUVECs were seeded in the microfluidic chip and cultured under perfusion until they formed a confluent parent vessel. The cells were activated by generating a gradient of AFs, which promoted EC migration and formation of angiogenic sprouts.

## 2 Materials and Methods

### 2.1 Chip Fabrication

The developed organ‐on‐a‐chip (OOC) system consists of a chip base and a glass slide. Subsequently, microstructures were printed inside the chip to finalize the design. The chip base was fabricated using a SLA printer (Form 3B+, Formlabs, USA) with BioMed Clear resin (Formlabs, USA). The printed chip underwent development and UV‐post‐curing as follows. The chip was immersed in isopropyl alcohol (IPA, >99%, Merck, Germany) for 15 min (Mercury X Wash, Elegoo, China). It was then air‐dried, soaked in fresh IPA for an additional 5 min, and air‐dried again. Afterwards, the chip was post‐cured (Form Cure, Formlabs, USA) for 30 min at 60°C.

The glass slide (21 × 26 mm #1 cover slip, Epredia, USA) was silanized with mercaptopropyltrimethoxysilane (175617, 95%, MPTMS, Merck, Germany) as following. First, the glass surface was activated with air plasma (Piezo Brush PZ3, Relyon Plasma, Germany) for 30 s at a less than 5 mm treatment distance. Afterward, the glass slide was immersed in a 10% MPTMS in absolute ethanol solution (>99%, Merck, Germany) for 1 h at RT. The silanized slide was rinsed with deionized water (MilliQ) and dried for 1 h at 60°C.

Double‐sided silicone adhesive tape (ARclad 7876, Adhesives Research, USA) was cut with a plotter (Cameo 3, Silhouette, USA) to match the shape of the microfluidic channels and applied between the glass and chip surfaces. Prior to this, both the glass and chip sides were plasma‐treated (Piezo Brush PZ3, Relyon Plasma, Germany) for 30 s at a distance of less than 5 mm. The bonded chip was then kept under pressure for 17 h at RT.

The process of preparing design files for 2PP‐printing involves creation of the 3D model using Computer Aided Design (CAD) software (Solid Edge, Siemens, Germany) and the use of slicing software (Bio2Print, LaVision BioTec, Germany) to convert the designed model into a format suitable for the 2PP printer (modified TriM Scope Matrix 2‐photon Microscope, LaVision BioTec, Germany). The printing system employs a Ti:Sapphire laser (Coherent, Chameleon Vision II, USA) with a tunable wavelength range of 680–1080 nm, delivering 140 fs pulses at an 80 MHz repetition rate.

The printing process was conducted as follows. First, the assembled chip was filled with a photosensitive resin containing precursor poly‐((D,L)‐lactide‐*co‐ε*‐caprolactone) dimethacrylate (LCM, iba Heiligenstadt, Germany) with six lactide and four caprolactone building blocks per molecule and 95% degree of methacrylation supplemented with 1% photoinitiator 2,5‐bis[4‐[N,N‐bis‐[2‐(acetyloxy)ethyl]phenyl]‐methylene]‐(2E,5E)‐cyclopentanone (BA740, iba Heiligenstadt, Germany). Next, the chip was positioned in the 2PP printing system. Microstructures were fabricated at a writing speed of 150 mm/s, with a laser power of 80 mW, a distance between layers of 30 µm, and a distance between printed lines of 2 µm. After printing, the quality of the structures was assessed using two‐photon excitation microscopy within the same system. The power was reduced to less than 1 mW to avoid further polymerization. Afterward, the unpolymerized LCM‐BA740 material was removed by development with OrmoDev (Micro resist technology, Germany) for 15 h. Finally, OrmoDev was removed from the channels. The chip was dried and placed in a UV curing machine (Form Cure, Formlabs, USA) for 12 h for post‐curing using light of 405 nm wavelength.

### 2.2 LCM‐BA740 Printing Parameters Optimization

To determine the optimal printing parameters for the LCM‐BA740 material, a parameter sweep was performed. To prepare a test sample, a 1 mm thick silicone film frame was placed on a silanized glass to restrict material spreading over the glass surface. LCM‐BA740 was cast and heated up to 70°C for 20 min to ensure homogeneous resin distribution and absence of air bubbles that could affect the printing process. Printing was performed at an 800 nm wavelength using a 4× objective on an inverted stage. A square pattern with dimensions of 200 × 200 µm was selected for the printing design. The parametric sweep included power levels ranging from 5 to 180 mW and three distinct categories of writing speed: slow (from 0.4 to 4.2 mm/s with a step width of 0.2 mm/s), medium (from 3 to 60 mm/s with a step width of 3 mm/s), and fast (from 220 to 400 mm/s with a step width of 10 mm/s). Following the printing process, the samples were developed with OrmoDev (Micro Resist Technology, Germany) to remove any non‐polymerized material. Structures that remained adhered to the silanized glass after development were imaged using two‐photon excitation microscopy with a 20X objective on an upright stage at 900 nm and 60 mW. The images obtained were y,z projections, providing information about the printed structures' height and their overall integrity. Data analysis focused on correlating the height of the printed structures with the tested power levels and writing speeds. Additionally, adherence of the structures to the substrate was analyzed.

The next parameter optimization experiment involved printing 100 × 100 µm structures with 300 µm height using varying power from 0 to 185 mW and variable distance between layers from 10 to 100 µm. This process was repeated for four different printing speeds starting from 50 mm/s and up to 200 mm/s using a 50 mm/s step width. The printing was done at 800 nm wavelength using a 4× objective. After development with OrmoDev, y,z projections of the polymerized structures were obtained using two‐photon excitation microscopy at 900 nm wavelength, 60 mW power and 20× objective. The analysis of the results focused on investigating the effects of laser power and layer spacing on the height and integrity of the printed structures.

### 2.3 Cell Culture

Human umbilical vein endothelial cells expressing green fluorescent protein (cAP‐0001GFP, GFP‐HUVECs, Angio‐Proteomie, USA) were cultured in T175 flasks (353112, Falcon, Corning, USA) using endothelial cell medium. Endothelial Cell Growth Medium 2 (C‐22011, PromoCell, Germany) supplemented with 1% penicillin/streptomycin (15140122, Thermo Fisher, USA) was used. Cells were split every 5 days in a 1:3 ratio and seeded in chips at passage 6. For cell detachment, a T‐175 flask was washed 1× with 10 mL PBS and filled with 7 mL accutase (1 mL per 25 cm^2^) (L0950, Biowest, USA). For all experiments, settings of the humidified incubator were set to 37°C and 5% CO_2_. The cell culture was carried out in a mycoplasma‐free lab and cells were tested negatively for mycoplasma before being used in any experiments.

### 2.4 LCM‐BA740 Biocompatibility Test

LCM‐BA740 discs (5 mm diameter, 1 mm height) were 2PP‐printed and afterwards washed for 1 week in IPA followed by another week in DI‐water at RT. The washed discs were dried and sterilized for 10 min with oxygen plasma containing 50% hydrogen fluoride. These sterile discs were placed in 24‐well tissue culture polystyrene (TCPS) plates (CLS351147, Falcon, Corning, USA) and were either seeded with GFP‐HUVECs directly or first coated with 10 µg/cm^2^ human plasma fibronectin (F1056, 95%, Merck, Germany) aqueous solution for 2 h at RT. Cells were seeded at a density of 10,000 cells/cm^2^. Medium exchange was performed every 3 days. Cell growth was monitored every day using fluorescent microscopy (EVOS M5000 Imaging System, Invitrogen, USA).

### 2.5 Chip Preparation for Cell Culture

The chip was immersed in DI‐water for 1 week to remove unreacted resin components, then dried and sterilized for 10 min using oxygen plasma containing 50% hydrogen fluoride. The oxygen pressure was set to 0.22 mbar (Tetra 30 plasma system, Diener Electronic, Germany).

The sterilized chip was filled with an extracellular matrix (ECM) gel according to the following protocol [20]: 100 µL of 4 mg/mL ECM gel solution was prepared on ice by carefully mixing 10 µL of 1 M (4‐(2‐hydroxyethyl)‐1‐piperazineethanesulfonic acid) buffer solution (HEPES, L0180, Biowest, USA) with 10 µL of 37 g/L NaHCO3 (S5761, Merck, Germany). Afterward, 80 µL of 5 mg/mL Cultrex 3D Culture Matrix Rat Collagen I (3447‐020‐01, Biotechne, USA) was added to the solution and mixed by pipetting, avoiding air bubble formation. This gel was kept on ice and used within 10 min. Inlets and outlets of the side Channels 1 and 3 (Figure 6A) were closed with silicone caps, and 10 µL of ECM solution was pipetted to Channel 2. The chip was placed in the humidified incubator (51032720, Heracell, ThermoFisher, USA) for 15 min for polymerization.

Side Channels 1 and 3 (Figure 6A) were coated with 10 µg/cm^2^ human plasma fibronectin (F1056, 95%, Merck, Germany) aqueous solution for 2 h at RT. Afterward, side Channels 1 and 3 were washed 1× with PBS (392‐0442, Avantor, USA) and filled with cell culture medium (Endothelial Cell Growth Medium 2, C‐22011, PromoCell, Germany).

### 2.6 Angiogenesis‐on‐a‐Chip Assay

GFP‐HUVECs were collected from the T‐175 flask and resuspended in endothelial cell medium at a concentration of 20× 10^6^ cells/mL. 10 µL of cell suspension was added to Channel 1 (Figure 6A) by pipetting. The chip was wrapped with parafilm (P7668, Merck, Germany), and placed upside down in a petri dish (P5731, Merck, Germany) in the incubator. This petri dish was wrapped with parafilm to prevent medium evaporation. After 30 min of incubation, the chip was turned by 180° and left for another 3 h in the incubator. At the next step, unattached cells were removed from Channel 1 (Figure 7A) by medium exchange. Then, Channel 1 was connected to 2 µL/min perfusion with a peristaltic pump (MFLX78001‐82‐EU, Reglo ICC Digital Pump, Masterflex, USA). The connection of the microfluidic chip to the peristaltic pump was established using tubing, where Tygon ND‐100‐80 tubing (AAD04103, Saint‐Gobain Life Sciences, France) served as the main tubing, and Tygon S3 E‐LFL tubing with three stops (070603‐05‐ND, Saint‐Gobain Life Sciences, France) was inserted into the pump head. Both tubings were connected to each other using KDS2112P Weller needles, and the junctions were additionally sealed with medical‐grade silicone adhesive (MED1‐4213, NuSil, USA) to prevent any leakage. The tubing connection to the microfluidic chip was done with KDS2112P Weller needles.

Cells were cultured on a chip for 2 days until the formation of the confluent tube in Channel 1 occurred. The angiogenic cocktail was prepared by adding angiogenic factors (AFs) to the endothelial cell medium. The stock concentrations of the angiogenic components are:

‐ 100 µg/mL of recombinant human vascular endothelial growth factor‐165 (rhVEGF‐165, 100‐20, Peprotech, USA) in 0.1% bovine serum albumin (MACS BSA Stock Solution, 130‐091‐376, Miltenyi Biotec, Germany) dissolved in PBS;
‐ 10 µg/mL of phorbol 12‐myristate 13‐acetate (PMA, P1585, Merck, Germany) in 0.1% dimethyl sulfoxide (DMSO, >99.7%, D2650, Merck, Germany) dissolved in MilliQ water;
‐ 1 mM of sphingosine‐1‐phosphate (S1P, S9666, Merck, Germany) in 95% DMSO and 5% 1 M hydrochloric acid (HCl, 7647‐01‐0, Avantor, USA);
‐ 50 µg/mL of recombinant human basic fibroblast growth factor (rhFGFb, 100‐18B, Peprotech, USA) in 0.1% BSA dissolved in PBS;
‐ 100 µg/mL of recombinant human monocyte chemotactic protein‐1 (rhMCP‐1, 11343384, ImmunoTools, Germany) in 0.1% BSA dissolved in PBS;
‐ 100 µg/mL of recombinant human hepatocyte growth factor (rhHGF, 11343413, ImmunoTools, Germany) in 0.1% BSA dissolved in PBS.

The following volumes of stock solutions were added to 10 mL of medium: 3.75 µL of rhVEGF‐165, 37.5 µL of PMA, 2.5 µL of S1P, 7.5 µL of rhFGFb, 3.75 µL of rhMCP‐1, and 3.75 µL of rhHGF.

On Day 2 of GFP‐HUVECs cultivation in Channel 1, the medium in Channel 3 was replaced by the angiogenic cocktail. Channel 1 was perfused at a rate of 2 µL/min using a peristaltic pump (MFLX78001‐82‐EU, Reglo ICC Digital Pump, Masterflex, USA), whereas Channel 3 was perfused in the same direction at a rate of 1 µL/min. The resulting sprout formation was monitored using a fluorescent microscope (EVOS M5000 Imaging System, Invitrogen, USA).

### 2.7 Immunocytochemistry Staining on a Microfluidic Chip

Channels 1 and 3 on the chip were washed with PBS containing calcium and magnesium (PBS++, Cytiva, SH30264.01, USA). Afterward, Channels 1 and 3 were filled with 10 µL of 4% paraformaldehyde in PBS (PFA, J19943.K2, Thermo Fisher, USA) each and incubated at room temperature (RT) for 10 min. Starting from this step, the chip was protected from light. The 4% PFA was removed from the channels, and they were washed three times for 5 min with PBS++. A permeabilization buffer (PB) was prepared, consisting of 0.2% Triton‐X‐100 (X‐100, Merck, Germany) and 0.5% bovine serum albumin in PBS (MACS BSA Stock Solution, 130‐091‐376, Miltenyi Biotec, Germany). The primary antibody (CD144, anti‐human, REA, pure, Miltenyi Biotec, Germany) was diluted 1:400 in PB. 10 µL of the primary antibody solution was pipetted into each channel and the chip was stored overnight at 6°C. Afterward, the channels were washed for 10 min for three times with 10 µL of AutoMACS Running Buffer (130‐091‐221, Miltenyi Biotec, Germany). The secondary antibody (Alexa Fluor 647, Goat anti‐Human IgG (H+L), Molecular Probes, USA) was diluted 1:400 and then filled into the chip, with 10 µL of the secondary antibody per channel, for 2 h at RT. Then, the channels were washed for 10 min three times with 10 µL of AutoMACS Running Buffer. The stained chip was filled with AutoMACS Running Buffer and stored at 6°C. Images of the stained cells were taken using a fluorescent microscope (EVOS M5000 Imaging System, Invitrogen, USA).

## 3 Results

An important step in organ‐on‐a‐chip (OOC) systems involves establishing 3D vascular networks, which are crucial for nutrient transport, waste removal, and the overall functionality of the engineered tissues. Therefore, our goal was to develop a versatile microfluidic chip tailored for vasculature‐on‐a‐chip applications, which could serve as the basis for more complex 3D in vitro models. The main part of the chip was manufactured with one‐photon printing (stereolithography, SLA), which is a perfect technique for fast production of larger structures at low costs. To further refine the chip, we used 2‐photon polymerization (2PP) technology, which enabled the printing of microstructures directly within the chip. Specifically, we designed the following microstructures that are essential for the chip's functionality:

1. The pillar array with a distinct border to separate the channels, ensuring proper fluid flow and compartmentalization (Figure 2A(I)).
2. The contour structure effectively seals the channels, preventing leaks and maintaining the integrity of the microfluidic environment (Figure 2A(II)).

The vasculature‐on‐a‐chip system was successfully seeded with human umbilical vein endothelial cells expressing green fluorescent protein (GFP‐HUVECs). By Day 2, GFP‐HUVECs had successfully formed a parent vessel within Channel 1. When angiogenic factors (AFs) were introduced into Channel 3, the endothelial cells (ECs) responded by migrating into the extracellular matrix (ECM) within Channel 2, where they formed angiogenic sprouts (Figure 9). This demonstrates the chip's potential to support vascular formation, making it a powerful tool for tissue perfusion studies and future OOC systems.

### 3.1 Chip Fabrication

**FIGURE 1:**
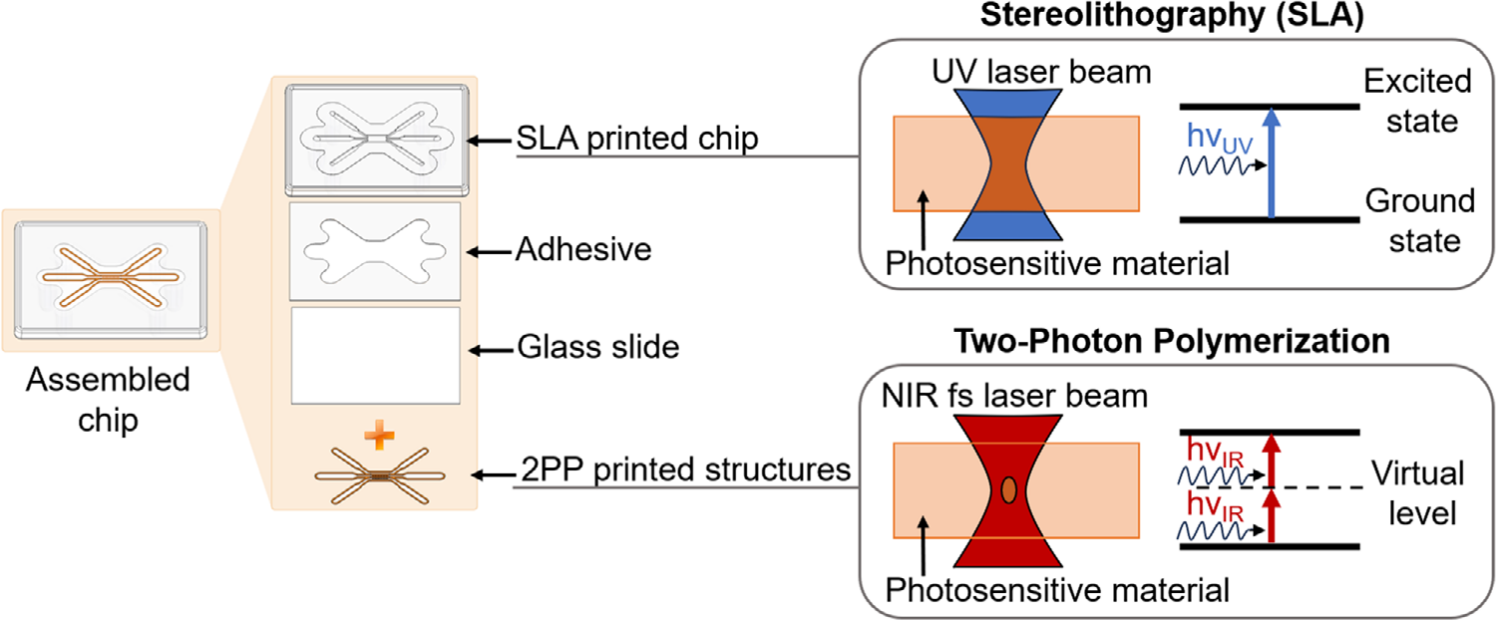
Left: schematic illustration of the chip assembly. Chip base is bonded to a glass slide with a double‐sided adhesive tape. Right: additive manufacturing techniques used for chip fabrication. Chip base was printed with stereolithography, microstructures were implemented by two‐photon polymerization printing directly in the assembled chip.

Our approach to chip fabrication involves a two‐step process, combining the advantages of SLA to manufacture the chip base and 2PP technology for finalizing the chip by printing the microstructures and ensuring reliable bonding in challenging areas (Figure 1). For instance, while glue generally provides good bonding in easily accessible areas, it can overflow in case of narrow or closely positioned channels, and double‐sided adhesive tape may cause leakage due to misalignment or resolution limitations of the plotter, making it unsuitable for complex designs. Additionally, glues and tapes often lack sufficient resistance to development solvents, which can result in chip damage. Thus, combining the strengths of 2PP printing—such as precision, high resolution, and solvent resistance—with the advantages of traditional gluing methods, which enable fast and easy bonding of larger areas with simple geometries, presents a promising approach to optimize the bonding process and enhance the chip quality. Furthermore, our approach offers design flexibility allowing to use the main chip for multiple applications depending on the design of the 2PP‐printed microstructures.

SLA technology, which was used to produce the chip base, is the preferred method for creating microfluidic components due to its resolution and reproducibility. SLA operates on the principle of photopolymerization, where the energy of a single photon induces the polymerization of the photosensitive material (Figure 1). This method provides sufficient resolution in a µm‐range and offers fast printing speed [21]. BioMed Clear resin (Formlabs, USA) was chosen as a printing material due to its transparency and biocompatibility.

The glass slide serves as the bottom layer of the chip. Silanization is performed to promote adhesion of 2PP printed structures. The main part of the chip is bonded to the glass slide with pressure‐sensitive adhesive tape. This adhesive tape demonstrated superior bonding strength, biocompatibility, and resistance to development solutions as compared to other gluing methods such as UV glue, silicone glue, and temperature‐sensitive tape. The assembled chip is filled with 2PP resin for further printing of the microstructures.

In contrast to SLA, the 2PP technique requires simultaneous absorption of two photons to reach the excited state, which occurs exclusively at the center of a focused laser beam (Figure 1). Due to the non‐linearity of the process, this provides sub‐100‐nm resolution together with a spatial control allowing for fabrication of microstructures with intricate geometries directly inside OOC devices [21].

**FIGURE 2:**
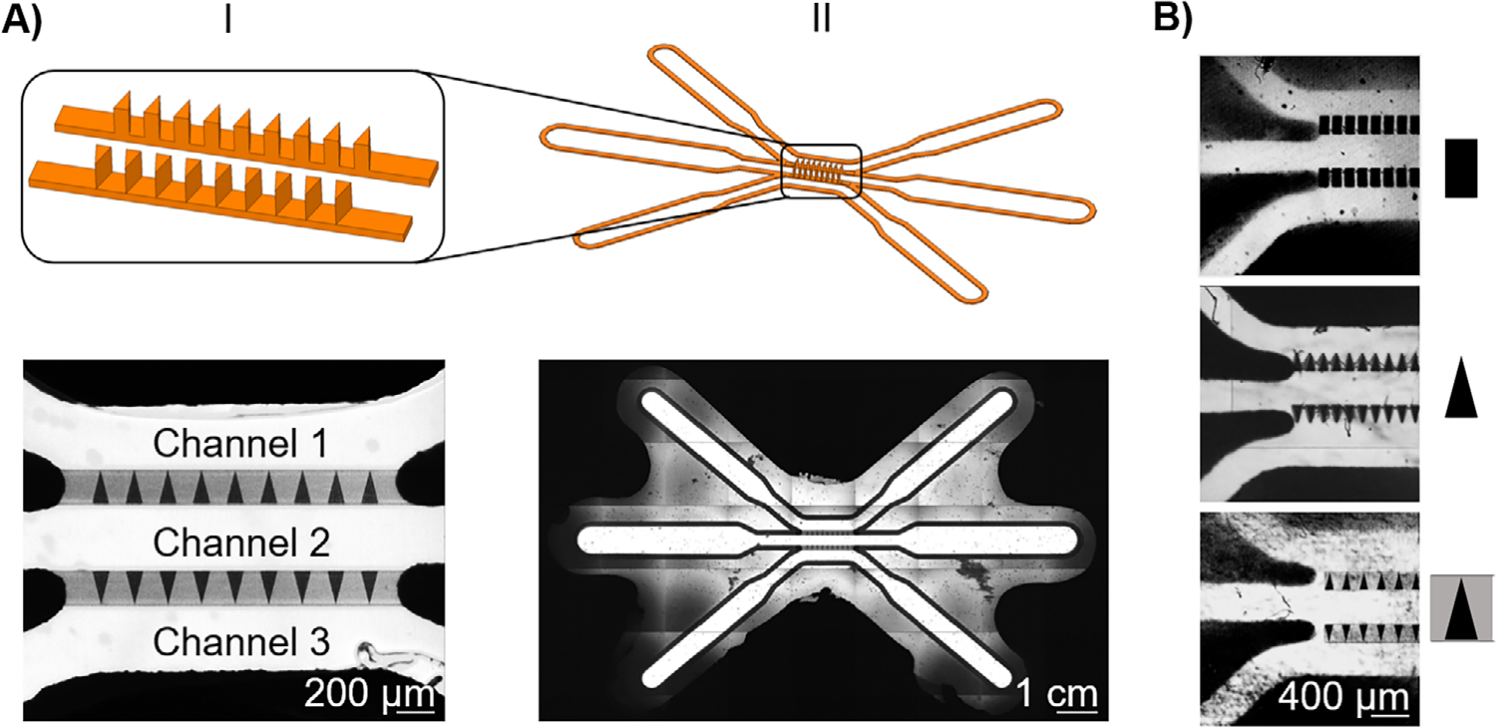
Structures printed with two‐photon polymerization. (A) Left: CAD model of triangular pillar array with a border and corresponding result of a 3D print in a microfluidic chip in the lower part. Right: CAD model of a sealing contour and corresponding result of a 3D print in a microfluidic chip in the lower part. (B) Various designs of barrier structures between the channels. From top to bottom: rectangular pillars without a border, triangular pillars without a border, triangular pillars with a border.

The initial structure fabricated using the 2PP‐method was an array of pillars (Figure 2A(I)). The triangular shape of the pillars with their apices facing away from Channel 2 facilitated cell migration while retaining the gel within central Channel 2. Additionally, since the gel fills the spaces between the pillars, cells have increased contact with the gel rather than the printing material in Channels 1 and 3, where cells are cultured, promoting cell migration inside the gel.

In the second design iteration (Figure 2A(II)), a sealing contour was added to achieve reliable attachment between the glass and the chip base, particularly in areas that are difficult to reach with standard gluing techniques. Another function of the contour is the protection of the channels from aggressive solvents during the development step. Therefore, the addition of the contour improves the integrity and functionality of the microfluidic chip by preventing leakage and ensuring robust bonding. Figure 2B illustrates examples of pillar designs, which can, for instance, differ in shape (rectangular or triangular), or exhibit a border. The border facilitates gel filling without the need for additional surface modifications, such as hydrophobization of the side Channels 1 and 3 and hydrophilization of the Channel 2. The final design features a triangular pillar array with a border (Figure 2B, bottom), as the triangular pillar shape enhances cell‐ECM contact, while the border helps retain the ECM gel within the central channel.

Our innovative two‐step chip fabrication process combines the strengths of SLA and 2PP technologies, resulting in high‐quality bonding and flexible design options for diverse applications. This method not only ensures robust and leak‐proof microfluidic chips but also supports cell migration by optimizing microstructure designs, greatly contributing to the development of OOC devices.

### 3.2 LCM‐BA740 Printing Parameters Optimization

**FIGURE 3:**
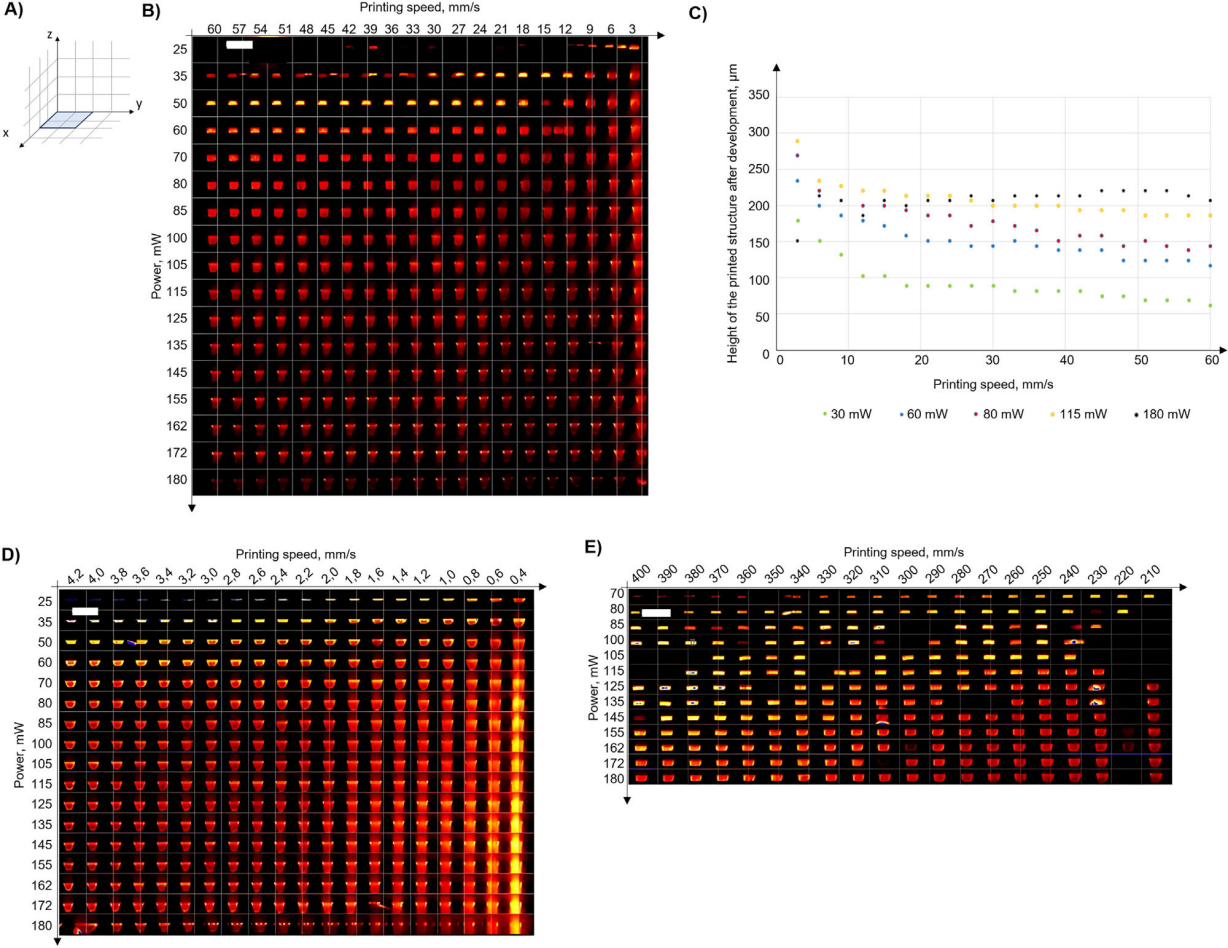
(A) Schematic illustration of the structure for printing: a 200 × 200 µm square was printed in the XY plane at Z = 0. (B) Two‐photon excitation microscopy image of the printed and developed structures for the medium printing speed range from 3 to 60 mm/s. (C) Correlation between the height of the printed structures after development for the medium printing speed range from 3 to 60 mm/s depending on the used laser power. (D) Two‐photon excitation microscopy image of the printed structures after development for the low printing speed range from 0.4 to 4.2 mm/s. (E) Two‐photon excitation microscopy image of the printed structures after development for the high printing speed range from 210 to 400 mm/s. Scale bar (top left): 450 µm.

To determine optimal printing parameters set for 2PP printing with the LCM‐BA740 material, a parameter sweep was conducted by varying laser power and writing speed. Three sets of printing tests were conducted, representing medium (Figure 3A–C), low (Figure 3A,D), and high (Figure 3A,E) printing speeds, to analyze the material's behavior under different laser power levels. The printed structures were subjected to a development procedure and subsequently imaged using two photon excitation microscopy.

Adhesion of the printed structures to the silanized glass was strongly influenced by the applied laser power. The results revealed that for printing speeds below 12 mm/s, a minimum laser power of 25 mW or higher was required to ensure that the printed structures remained attached to the glass slide after the development step (Figure 3B). For intermediate printing speeds, ranging from 12 to 60 mm/s, a minimum laser power of 35 mW was necessary to observe the structures post‐development (Figure 3B). Finally, at high printing speeds between 210 and 400 mm/s, at least 70 mW of laser power for the printed structures to withstand the development process (Figure 3E).

Interestingly, lower power levels did activate the material polymerization, as printed structures were visible immediately after printing and prior to the development process (data not shown). However, these structures did not survive the development step, likely due to insufficient crosslinking within the material, resulting in weak bonding between the material and the glass substrate. Low power levels may not provide enough energy to fully initiate and propagate the polymerization reaction, resulting in partially cured structures. During the development process, these weakly crosslinked regions dissolve or break apart when exposed to solvents. This suggests that a threshold power level is necessary to achieve robust polymerization and produce stable structures capable of withstanding subsequent processing steps.

Another interesting observation was the variation of height of the 200 × 200 µm structures printed as a single layer (Figure 3A). The height of these squares increased with both higher laser power and slower writing speed. This can be attributed to the greater energy deposition at higher powers or slower speeds, which promotes enhanced cross‐linking and polymerization. Conversely, faster writing speeds or lower laser powers result in reduced energy deposition, leading to less material polymerization and, consequently, structures of less height.

The relationship between the height of the printed squares, writing speed, and power is illustrated in Figure 3C. This data highlights the critical influence of these parameters on achieving uniform and stable structures, underscoring the need for careful optimization to balance resolution, adhesion, and printing efficiency. Based on the experimental results, structures printed with a laser power of 80 mW and a printing speed of 50 mm/s could support a layer spacing of up to 150 µm, which was further examined in subsequent printing tests.

**FIGURE 4:**
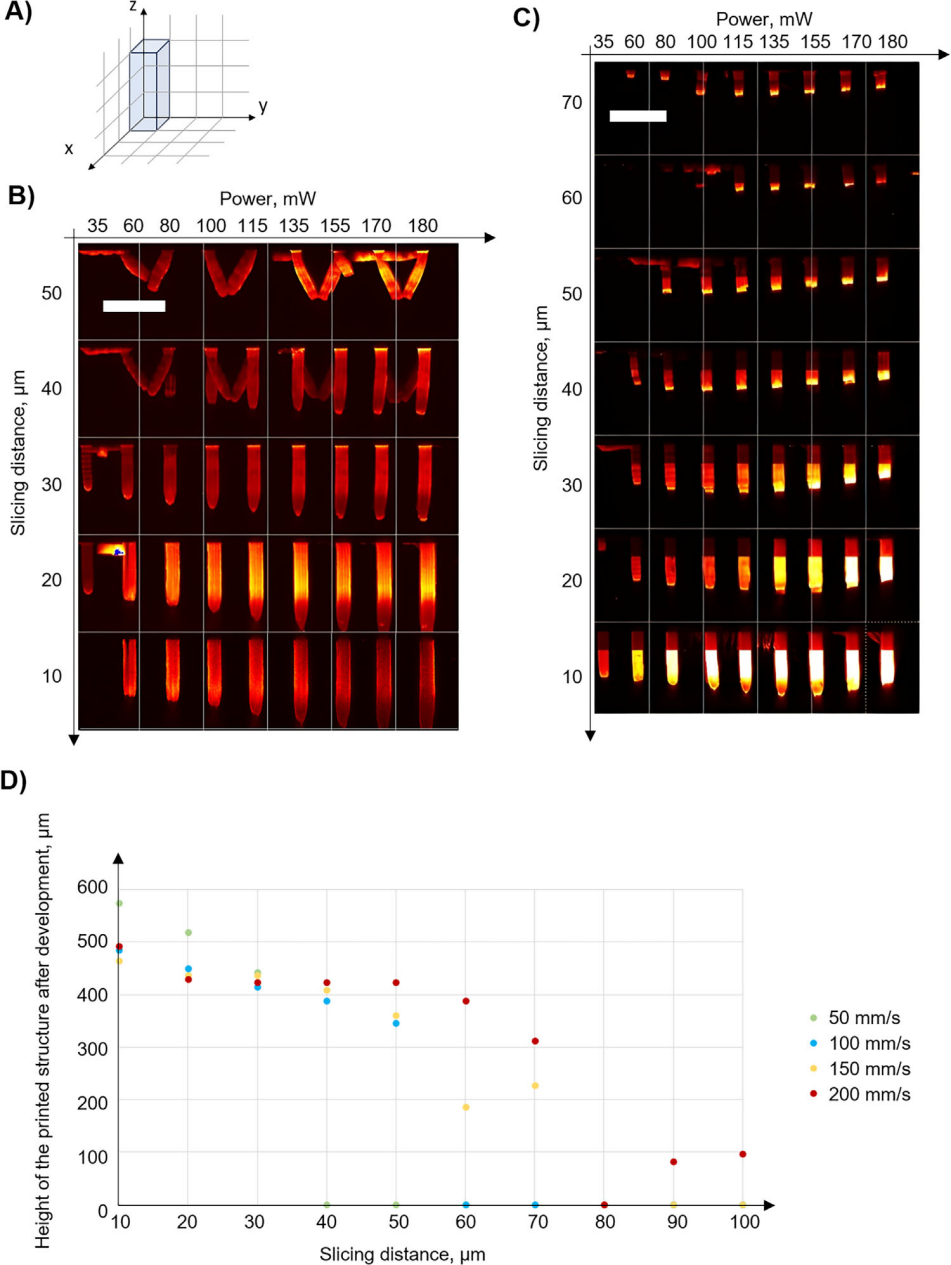
(A) Schematic illustration of the structure for printing: a 100 × 100 × 300 µm cuboid was printed along the X, Y, and Z axes with the respective dimensions. (B) Two‐photon excitation microscopy image of the printed and developed structures at the 100 mm/s printing speed. (C) Two‐photon excitation microscopy image of the printed structures after development for the 200 mm/s printing speed. (D) Correlation between the height of the printed structures after development and the slicing distance for the following printing speeds. 50, 100, 150, and 200 mm/s. Structures were printed with 80 mW laser power. Scale bar (top left): 450 µm.

The next experiment aimed to have a closer look on the influence of the layer spacing on the height and integrity of the printed structures. For this purpose, 100 × 100 µm structures with a height of 300 µm (Figure 4A) were sliced into layers with distances between them varying from 10 to 100 µm with a 10 µm step width and printed at different laser power settings. This process was repeated for different printing speeds. Figure 4B,C illustrates the results obtained for printing speeds of 100 and 200 mm/s, respectively.

As expected, printing with higher powers resulted in increased height of the printed structures. The minimum power required for the structures to be present after development was 35 mW. The height of the structures was inversely proportional to the spacing between layers. This effect could be explained by insufficient overlap between layers at slicing distances above 30 µm, resulting in a weaker structural cohesion, and the effects of shrinkage or deformation during development. Reducing the slicing distance increases the structure's height, likely due to over‐polymerization, resulting in excessive polymerization and contributing to unwanted vertical growth, leading to structural inaccuracies or defects. Therefore, it is essential to optimize the layer spacing to ensure strong and stable interlayer connections.

A detailed analysis was conducted on structures printed at 80 mW with printing speeds ranging from 50 to 200 mm/s, as these parameters were estimated to be optimal for fabricating the microfluidic chip. While it was initially expected that a layer spacing of up to 150 µm at a printing speed of 50 mm/s would ensure sufficient overlap and structural integrity of the printed and developed structures, this was not observed. Structural integrity was maintained only for layer spacings up to 30 µm across all tested speeds, with larger spacings resulting in reduced size, deformation, or complete loss of the cuboids (Figure 4D). Increasing laser power did not compensate for the loss of integrity caused by excessive layer spacing.

Based on these results, the final parameters for printing the microfluidic chips were established at 80 mW laser power, 150 mm/s printing speed, and 30 µm layer spacing. This parameter set enabled efficient and reliable printing of 3D microstructures within a reasonable timeframe.

### 3.3 LCM‐BA740 Biocompatibility Test

**FIGURE 5:**
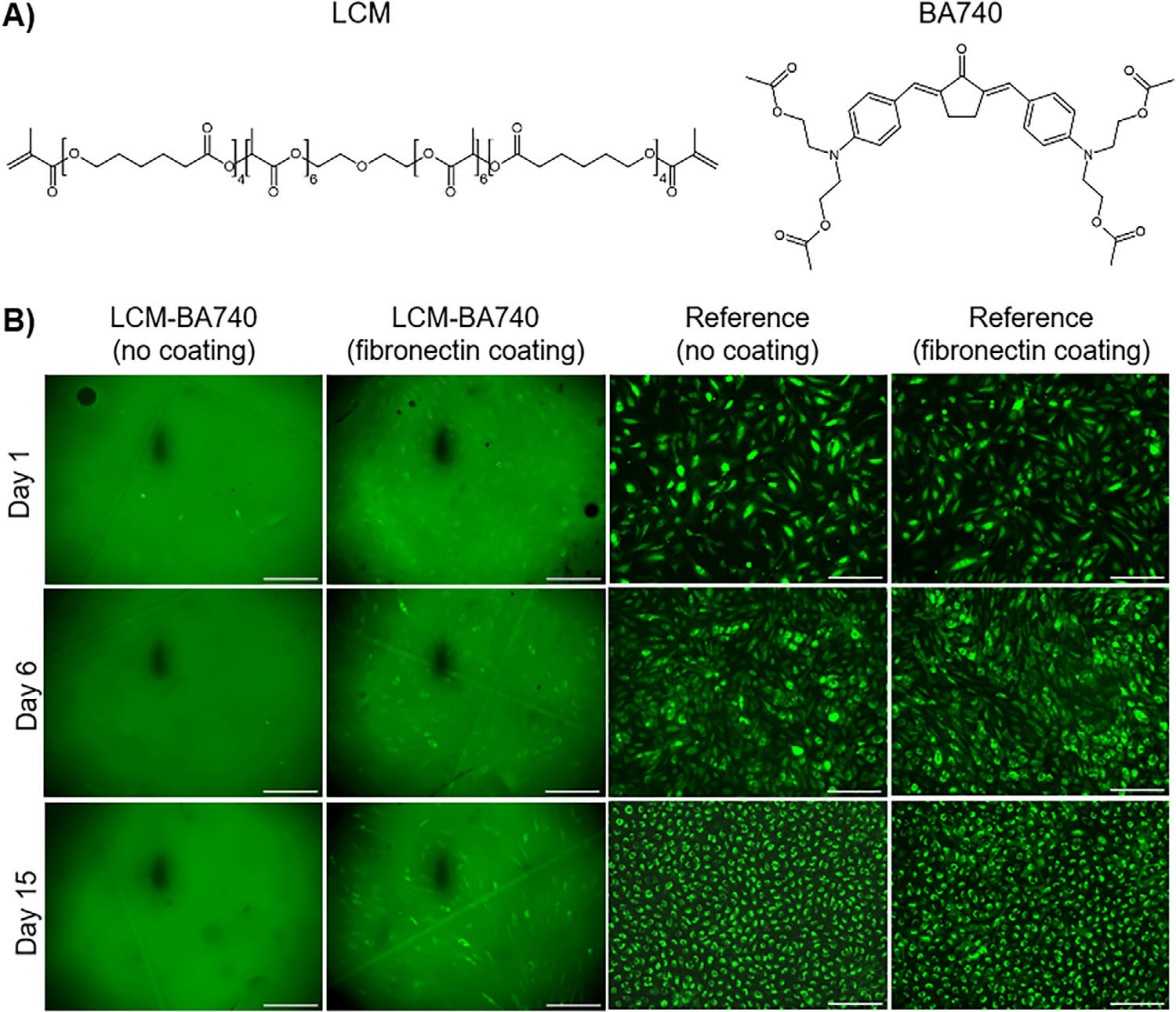
(A) Left: Chemical structure of the LCM precursor. Right: Chemical structure of the BA740 photoinitiator. (B) Enhanced GFP‐HUVECs (human umbilical vein endothelial cells expressing green fluorescent protein) adhesion on fibronectin‐biofunctionalized LCM‐BA740 discs in comparison to discs with no coating. As a reference, GFP‐HUVECs were cultured on tissue culture polystyrene plates, both with and without fibronectin coating. Fibronectin concentration: 10 µg/cm^2^. GFP‐HUVECs seeding density: 10,000 cells/cm^2^. Scale bar 250 µm.

One of the central aspects in the development of OOC systems is the selection of suitable materials. Synthetic biodegradable polymers provide adjustable mechanical stiffness, hydrophobicity and degradation rates, enabling customization to match the requirements of specific OOC applications. The LCM copolymer used in this work (Figure 5A) enables the adjustment of material degradation properties, stiffness, and hydrophilicity by varying the molar ratio of lactide to caprolactone units [16].

**FIGURE 6:**
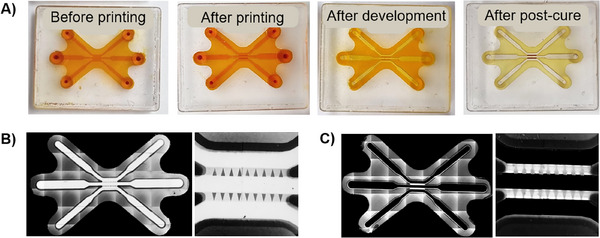
(A) Chip structure printing process by two‐photon polymerization. From left to right: chip filled with LCM‐BA740 before two‐photon polymerization printing, chip after printing, chip after development, and chip after post‐cure. (B) Two‐photon excitation microscopy images of the chip after printing. (C) Two‐photon excitation microscopy images of the chip after development.

Biocompatibility is a crucial aspect in the selection of materials for OOC fabrication, ensuring minimal cytotoxicity and promoting cell adhesion, proliferation, and differentiation. In photosensitive resins, components that could be cytotoxic are mainly the photoinitiator and unreacted monomers [22]. BA740 is a photoinitiator specifically designed for 2PP, giving the material a bright orange color (Figure 6A). BA740 was used at a concentration of 1%. Fully polymerized LCM‐BA740 demonstrates reduced color intensity changing from bright orange to dark yellow, allowing for visually estimating the degree of polymerization (Figure 6A). To minimize possible cytotoxic effects, we optimized the postprocessing routine by focusing on steps such as development, post‐curing, and washing, as described in Sections 2.1 and 2.4.

Even when showing suitable biocompatibility, synthetic polymers often lack cell‐adhesive motifs and bioactive cues, requiring additional biofunctionalization to enhance cell‐material interactions within OOC systems [23].

Before starting cell culture in chips, we first verified on LCM‐BA740 discs whether biofunctionalization of LCM‐BA740 with fibronectin effectively supports attachment of GFP‐HUVECs. The results confirmed that coating LCM‐BA740 with 10 µg/cm^2^ of fibronectin facilitates cell adherence in comparison to uncoated samples. Figure 5B illustrates that for the uncoated samples, the fluorescence signal primarily originates from the material's autofluorescence, while the fibronectin‐coated samples exhibit a visible monolayer of cells along with the fluorescent background. On the reference TCPS plate, cells formed a dense layer under both coated and uncoated conditions.

The selection of LCM‐BA740 as a material for OOC systems combines good biocompatibility and effective polymerization. Our findings demonstrate that fibronectin coating enhances cell‐material interactions, confirming the material's suitability for OOC applications.

### 3.4 Application and Analysis

**FIGURE 7:**
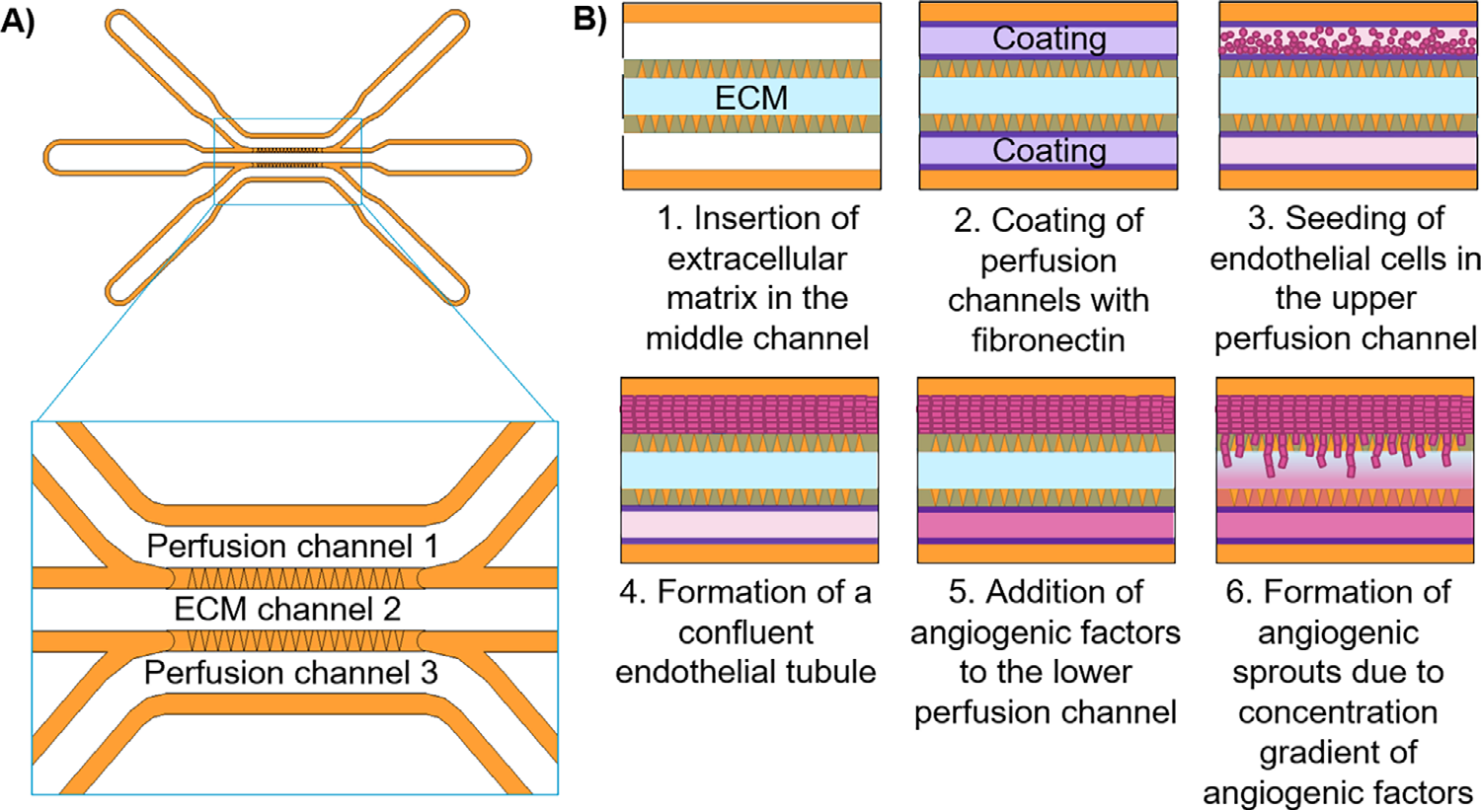
(A) Microfluidic chip design: showing channels and their functionality. In the perfused Channel 1 (2 µL/min perfusion rate), formation of parent vessel takes place, Channel 2 (no perfusion) contains extracellular matrix, whereas Channel 3 (1 µL/min perfusion rate) is used for introducing angiogenic factors. (B) Sequence of the angiogenesis assay. Channel 2 of the chip is filled with extracellular matrix. Channel 1 is seeded with endothelial cells. When endothelial cells form a confluent tube, angiogenic factors are introduced into the Channel 3. That leads to the formation of a gradient of angiogenic factors in the extracellular matrix and promotes angiogenesis.

The microfluidic chip presented in this work is designed for researchers to explore the complex world of vascular biology. This chip consists of three channels (Figure 7A) that are used to study angiogenesis—the formation of new blood vessels from the existing parent vessel [24]. The middle channel (Channel 2) of the chip is filled with ECM components, while the side channels (Channels 1 and 3) are used for cell cultivation under perfusion.

The angiogenesis assay is performed as depicted in Figure 7B. First, Channel 2 is filled with Collagen I and then allowed to polymerize. The selection of Collagen I as the ECM component is justified for several reasons. First, collagen is present in many tissues, including vasculature, thus closely resembling the natural environment for ECs. Second, Collagen I has binding sites for cell surface receptors, facilitating EC adhesion and migration within the ECM. Finally, Collagen I is biodegradable and can be remodeled by cells during the formation of new vascular structures [25]. Perfusion Channels 1 and 2 are biofunctionalized with fibronectin to support cell adhesion to the printing material. Perfusion Channel 1 is seeded with GFP‐HUVECs, which adhere to the fibronectin‐coated surface and proliferate under 2 µL/min perfusion until they form a confluent tube. Once the ECs have formed a parent blood vessel in Channel 1, a cocktail of AFs dissolved in cell culture medium is introduced into Channel 3. This creates a gradient of AFs within the ECM‐filled Channel 2 and stimulates sprouting formation and migration of ECs through the gel toward the opposite chip side, inducing angiogenesis.

**FIGURE 8:**
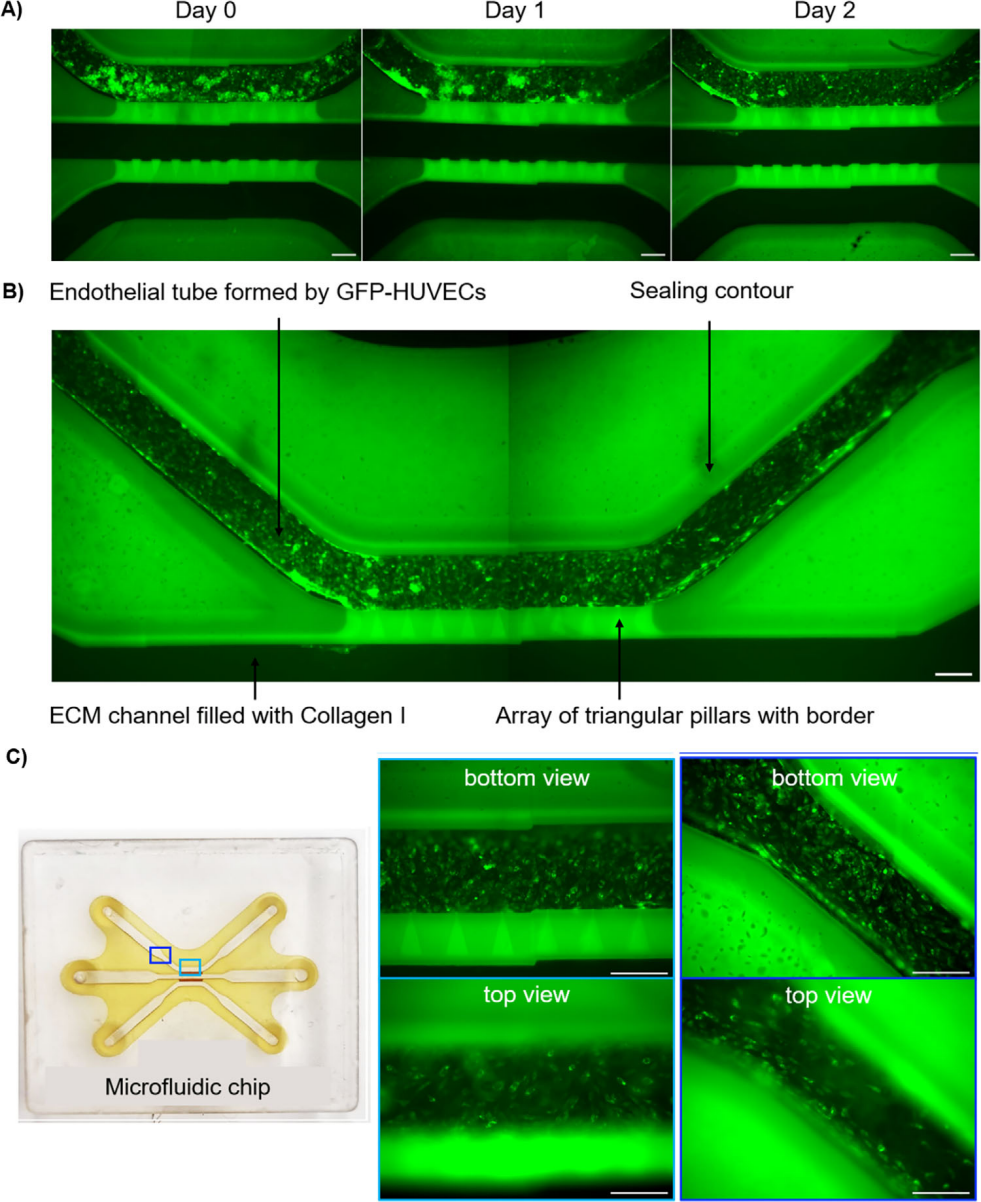
(A) Distribution of GFP‐HUVECs (human umbilical vein endothelial cells expressing green fluorescent protein) in Channel 1: cell clusters are washed out after 2 days of perfusion. (B) Image of the chip ready for angiogenesis assay, showing a confluent endothelial tube in Channel 1. (C) Verification of GFP‐HUVECs layer confluency at different focal planes. The bottom and top views of the central and side channel areas confirmed that GFP‐HUVECs formed a confluent parent vessel. Scale bar 250 µm.

The first days following cell seeding are critical for the experiment's success. The objective is to achieve a confluent layer of ECs in Channel 1. Confluency of the parent vessel reduces the likelihood of vessel regression and increases the stability of the formed vascular sprouts. However, prolonged cultivation can lead to overly tight cell‐cell junctions, suppressing cell sprouting [26]. We determined that the most favorable duration for culturing GFP‐HUVECs in the chip prior to activation with AFs is 2 days, given a seeding density of 20 × 10^6^ cells/mL. Figure 8A illustrates that on Day 0, cells are attached, but clusters of unattached cells remain stuck in Channel 1. After 1 day of perfusion with cell culture medium at 2 µL/min, the number of these clusters starts to decrease, and by Day 2, the cell distribution becomes visibly homogeneous. Figure 8B shows a chip on Day 2, ready for the angiogenesis assay.

To verify that ECs have formed a tube on Day 2, images of the cell layer were taken at different focal planes, particularly at the top and bottom of Channel 1 (Figure 8C). The confluency of the cell layer was confirmed not only in the central part of the chip but throughout the entire channel. This indicates that the GFP‐HUVECs in the parent vessel are ready to be stimulated with AFs.

**FIGURE 9:**
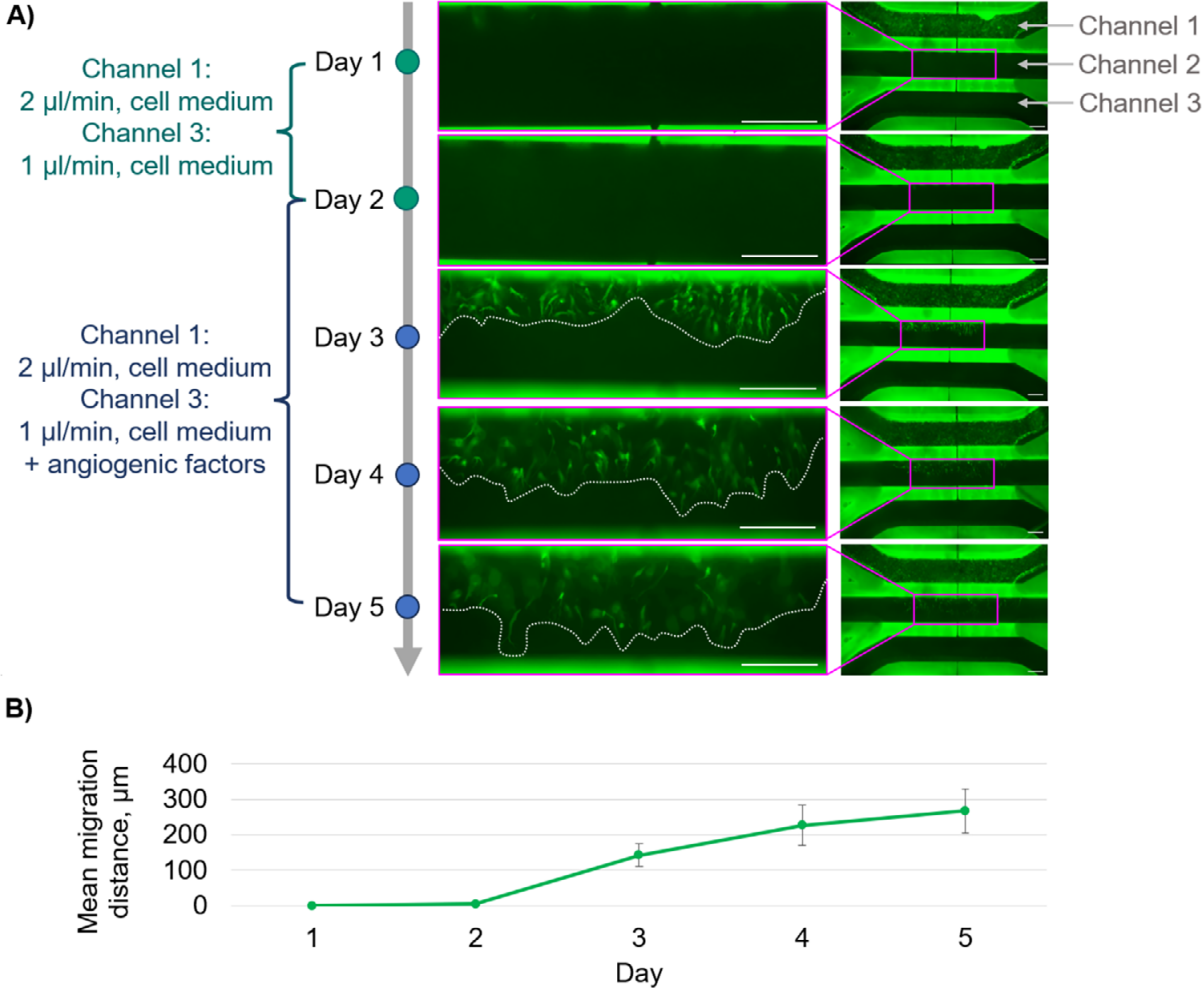
(A) Overview of the angiogenesis‐on‐a‐chip process, showing the initial formation of a confluent endothelial tube in Channel 1, the introduction of the angiogenic cocktail in Channel 3 on Day 2, resulting in cell migration and sprout formation by Day 3, and continued cell spreading in the gel over the following days. The white dotted line represents the migration boundary, indicating how far the cells have penetrated into the extracellular matrix gel. Scale bar: 250 µm. (B) Graphical representation of the mean endothelial cell migration distance into Channel 2 over the time period of 5 days.

Figure 9 provides an overview of the entire angiogenesis‐on‐a‐chip process, from the initial phase of achieving a confluent tube to the formation of angiogenic sprouts. During the first two days of parent vessel formation, no spontaneous cell migration was observed. On Day 2, an angiogenic cocktail was introduced to Channel 3, and by Day 3, cell migration following the concentration gradient and the successful formation of angiogenic sprouts were observed. An additional factor that supports the formation of angiogenic sprouts is the pressure gradient, since the perfusion rate of Channel 1 is twice that of Channel 3. In the following days, cells continued to migrate and spread further in the gel.

The angiogenic sprouts in Figure 9A are displayed in one focal plane, though more sprouts exist outside this focal plane. The background fluorescence of the LCM‐BA740 material allows for the clear identification of the channel structures, but limit at the same time the signal resolution to the same extend. Figure 9B presents a graphical representation of mean EC migration distance over time, providing a clear visualization of sprout growth dynamics throughout the culture period.

The microfluidic chip designed for vascular biology research successfully facilitates the study of angiogenesis by providing an optimal environment for endothelial cell cultivation and sprout formation. Our results demonstrate effective EC adhesion, proliferation, and migration, with clear formation of angiogenic sprouts after the introduction of AFs.

**FIGURE 10:**
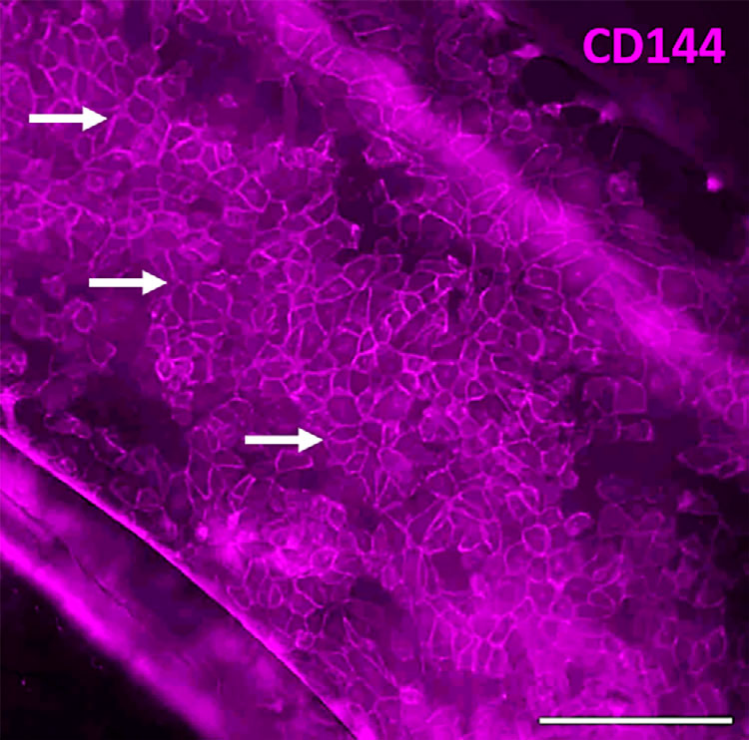
Immunofluorescence staining of GFP‐HUVECs (human umbilical vein endothelial cells expressing green fluorescent protein) with a CD144 membrane marker reveals its localization at intercellular junctions (white arrows), indicating the integrity of the cell layer within the microfluidic channel. Scale bar: 250 µm.

To assess the functionality of GFP‐HUVECs in the chip, we stained the cells with a CD144 membrane marker for ECs. To perform the staining we employed an indirect staining method, where the primary antibody was binding to CD144 and the secondary antibody was labelled with the fluorescent dye Alexa Fluor 647.The indirect staining approach yielded a stronger signal compared to the direct staining methods (data not shown). This signal amplification is due to the ability of several secondary antibodies to bind to a single primary antibody, which enhances signal detection [27]. All staining steps were conducted under conditions that minimized light exposure to prevent photobleaching. Indirect staining with the CD144 marker (Figure 10) effectively highlights its localization at intercellular junctions, confirming the integrity of the cell layer in our chip system.

While designed for angiogenesis, the chip can be adapted for other studies involving cell migration, ECM interactions, and the effects of various biochemical stimuli on cell behavior. This microfluidic chip is a powerful tool for researchers studying vascular biology, drug screening for angiogenesis inhibitors or promoters, and understanding the fundamental mechanisms of blood vessel formation.

## 4 Discussion

Tissue engineering faces significant challenges in creating functional organ‐on‐a‐chip (OOC) models. The choice of fabrication methods and materials is crucial for overcoming these challenges, as they directly impact the precision of structural features and the ability to generate viable vascular networks essential for tissue functionality [17, 28].

For the fabrication of our system, we used a combination of one‐ (SLA, stereolithography) and two‐photon polymerization (2PP) technologies. This approach offers a significant advantage over other methods, as 2PP printing enables the creation of highly precise and detailed microstructures with smooth surface finishes, while SLA printing provides high speed and flexibility in design and material selection for various applications [10, 21]. Moreover, both SLA and 2PP techniques are light‐based, which provides strong adhesion of the 2PP printed microstructures to the SLA manufactured chip base.

Materials used for 2PP printing are typically categorized into synthetic and natural polymers. Natural polymers offer the advantage of inherent biocompatibility and bioactivity, often mimicking the extracellular matrix (ECM) environment found in vivo. However, they show high batch‐to‐batch variation and may lack the mechanical properties needed for specific applications. In contrast, synthetic polymers stand out due to their well‐defined biophysical and biochemical properties, allowing for precise customization of mechanical strength, degradation rates, and other critical parameters. This predictability and tunability make synthetic polymers particularly advantageous for OOC applications where specific, consistent performance is essential [16]. The choice of the 2PP resin used in this work was given to a synthetic material, LCM‐BA740. This material demonstrated good mechanical stability, biocompatibility, and bonding of the printed structures to both silanized glass slides and SLA printed main chip parts. Furthermore, the resin allowed for immediate imaging of the printed structures using the same two‐photon system, saving time in the fabrication process.

As the LCM‐BA740's composition is still being refined, a key area for improvement is reducing its autofluorescence, which originates from the photoinitiator (PI) BA740. By lowering the PI concentration from 1% to 0.25%, autofluorescence was reduced by up to 60%.

Because LCM‐BA740 is a synthetic material, cell adherence had to be enhanced via surface treatment. To support cell adhesion in our system, we used fibronectin coating, which greatly mimics natural ECM and is fast and easy to apply. The next step could be to try alternative biofunctionalization methods mentioned in the literature [16]. For example, chemical grafting of N‐succinimidyl‐6‐(4′‐azido‐2′‐nitrophenylamino)‐hexanoate (sulfo‐SANPAH) linker molecules provides covalent bonding of biomolecules or cell adhesion sequences to the material surface. Another coating strategy involves electrostatic modification using the Layer‐by‐Layer (LbL) technique. By alternating layers of polycations (e.g., poly‐L‐lysine) and polyanions (e.g., heparin), a polyelectrolyte multilayer is formed, making the material suitable for cell growth [16]. These methods are more costly and complex processes than fibronectin coating but may offer higher effectiveness if further biofunctionalization optimization, such as for other cell types, is needed.

Vascularization is essential for advancing tissue engineering as it represents the first step toward developing viable organ models in the lab [17]. For instance, Kamm et al. have developed advanced organ‐on‐a‐chip models featuring functional 3D microvascular networks to study cancer cell extravasation. These models are typically fabricated using soft lithography, where polydimethylsiloxane (PDMS) is used to create microfluidic channels, and cell‐laden hydrogels are incorporated to mimic tissue structures [29]. However, a drawback of PDMS is its tendency to absorb small molecules, which can potentially interfere with experimental results [30]. Therefore, for the fabrication of our microfluidic system we used light‐based methods, which do not exhibit this issue, ensuring more reliable experimental outcomes. The developed chip effectively supports endothelial cell layer formation and angiogenesis, underscoring its utility in vascular biology research. The confluency of the parent vascular tube was proven by acquiring microscopic images at different focal planes along the z‐axis. An alternative method to ensure the readiness of the endothelial layer for angiogenesis assay would be a perfusion test with a dye. Good candidates for such a test are fluorescent dextrans or fluorescent microbeads. Additionally, they can be used later to estimate the perfusability of the formed angiogenic sprouts [20].

The presented angiogenesis‐on‐a‐chip system is designed to operate under low shear stress conditions. A flow rate of 2 µL/min was selected to mimic low‐shear (∼0.1 dyn/cm^2^) environments, which in vivo favor endothelial sprouting from regions with minimal or absent flow, such as occluded or blind‐ended vessels, while higher shear stresses stabilize mature vessels and inhibit new branch formation [31]. The presented angiogenesis‐on‐a‐chip system would be particularly suitable for screening anti‐angiogenic drugs for tumor growth suppression (e.g., Bortezomib [32] or Sunitinib [33]), or studying the effect of sphingosine 1‐phosphate (S1P) receptor agonists (e.g., FTY720 or VPC01091) [34] on endothelial sprout formation and vasculature development. Except for studying endothelial cell migration into the ECM, the system could also investigate migration potential of immune cells, for example, tumor or inflammation‐induced T‐cell extravasation [35]. Moreover, the microfluidic chip design allows to go beyond vasculature‐on‐a‐chip applications and could potentially be used as a gut‐on‐a‐chip model [36].

For studies aimed at endothelial cell alignment [37] or flow‐dependent gene expression [38], however, higher flow rates would be required. To sustain prolonged perfusion at elevated shear, further testing is needed, and the chip may require design modifications, such as enhanced protection of the ECM channel or development of advanced surface coatings to maintain robust cell adhesion under physiological shear conditions.

The developed system, compared to other angiogenesis‐on‐a‐chip platforms on the market (e.g., the Mimetas Organo Plate 3‐lane [20]), stands out due to such advancements as controlled perfusion, allowing to set up desired flow direction and flow rate, and adjustable design of barrier structures to meet specific user requirements. To further advance our system, we aim to increase the throughput and the degree of automation in the entire process management in the chip. Automation of the cell cultivation process will improve reproducibility and consistency, minimize manual handling errors, and enable more efficient experiment workflows. These advancements will broaden the potential applications of our angiogenesis‐on‐a‐chip system, making it a powerful tool for drug testing and for modeling physiological and/or pathophysiological processes.

## 5 Conclusion

In this study, we successfully developed a microfluidic chip designed for angiogenesis studies, incorporating a two‐step fabrication process that combines one‐ (SLA, stereolithography) and two‐photon polymerization (2PP) technologies. The SLA method provided a base for the chip, while 2PP allowed for the detailed printing of microstructures and enhanced bonding. The microstructures play a crucial role in ensuring leakage‐free perfusion and compartmentalization within the system. The 2PP resin LCM‐BA740, used in the chip fabrication, provides a unique combination of mechanical stability, biocompatibility, strong bonding capabilities, and customizable properties, making it a valuable material for advanced microfabrication and biomedical research.

Fibronectin coating of LCM‐BA740 facilitated endothelial cell (EC) attachment to the chip material and supported the maintenance of a flattened and elongated cell morphology, resembling the natural state of ECs in blood vessels. Moreover, fibronectin coating mimics the extracellular matrix (ECM) environment found in vivo, enhancing the physiological relevance of the system.

The functionality of the designed system was proven with an angiogenesis assay. Evaluation of our results indicates that our device can reliably support the formation and maintenance of a confluent EC layer, which is critical for effective angiogenesis. The introduction of angiogenic factors (AFs) under controlled conditions resulted in ECs sprouting, confirming the device's utility for vascular biology studies.

This angiogenesis‐on‐a‐chip system has high potential in advancing tissue engineering and regenerative medicine applications, particularly for studying angiogenesis under physiological and pathological conditions. For example, this system can be used to investigate abnormal angiogenesis, like the excessive vessel formation induced by tumor cells. Moreover, the angiogenesis‐on‐a‐chip system can be developed further into an organ‐on‐a‐chip (OOC) system, enabling culturing of vascularized spheroids, organoids, or tumoroids. The last ones would provide valuable insights into cancer progression and potential therapeutic interventions [18].

In conclusion, microfluidic chips for angiogenesis studies are becoming increasingly sophisticated and offering high levels of control over the cellular microenvironment. Future studies will implement methods of AI to analyze large datasets and to identify patterns in vessel formation under physiological and pathophysiological conditions, with this angiogenesis‐on‐a‐chip system serving as a promising platform for investigating and optimizing vascular development.

## Funding

The authors have nothing to report.

## Conflicts of Interest

The authors declare no conflicts of interest.

## Data Availability

The data that support the findings of this study are openly available in Zenodo at http://doi.org/10.5281/zenodo.15412184. Link: https://zenodo.org/records/15412185. DOI: 10.5281/zenodo.15412184
